# It Doesn’t End There: Workplace Bullying, Work-to-Family Conflict, and Employee Well-Being in Korea

**DOI:** 10.3390/ijerph15071548

**Published:** 2018-07-22

**Authors:** Gyesook Yoo, Soomi Lee

**Affiliations:** 1 Department of Child and Family Studies, Kyung Hee University, Seoul 02447, Korea; dongrazi@khu.ac.kr; 2 Department of Biobehavioral Health, Pennsylvania State University, State College, PA 16802, USA

**Keywords:** workplace bullying, quality of life, occupational health, work-to-family conflict, Korean workplaces

## Abstract

Workplace bullying entails negative consequences on workers’ life. Yet, there is lack of research on workplace bullying in an Asian context. Moreover, less is known about the potential mechanisms linking workplace bullying and employee well-being. This study examined the associations between workplace bullying and Korean employees’ well-being (quality of life, occupational health) and whether the associations were mediated by work-to-family conflict. Cross-sectional data came from 307 workers in South Korea who were employed in healthcare, education, and banking industries. Analyses adjusted for industry, age, gender, education, marital status, and work hours. Employees who had more exposure to workplace bullying reported lower levels of quality of life and occupational health. These associations were mediated by work-to-family conflict, such that more exposure to workplace bullying was associated with greater work-to-family conflict, which, in turn, was associated with lower levels of quality of life and occupational health. These mediating pathways were consistent across the three industries. Korean employees who experience more workplace bullying may bring unfinished work stress to the home (thus greater work-to-family conflict), which impairs their well-being. Future research may need to consider the role of work-to-family conflict when targeting to reduce the negative consequences of workplace bullying.

## 1. Introduction

Workplace bullying entails negative consequences on workers’ life, by exposing workers to negative acts of co-workers, supervisors or subordinates [1,2]. The prevalence of workplace bullying is high across nations [3] and it is becoming an increasingly serious issue in South Korea (Korea, hereafter) in recent years. The vast majority of Korean employees (87%) report they have experienced some form of bullying within the previous six months [4]. The rate of workplace bullying experiences is even higher among employees who work long hours and non-regular employees who may have job insecurity [5]. Workplace bullying may impair employees’ mental and physical health. However, there is lack of empirical research focusing on workplace bullying in Korea and its associations with Korean employees’ well-being. Moreover, less is known about potential mediating mechanisms linking workplace bullying and employee well-being [6].

Work-to-family conflict is a possible mediator between workplace bullying and employee well-being. Work-to-family conflict refers to time-based, strain-based, and behavior-based interrole conflict between mutually incompatible demands from work and family domains in some respect [7]. According to the work-family interface model [7,8,9], negative experiences and stressors from workplaces often spill over into employees’ personal and family life via work-to-family conflict [10,11,12,13]. Work-to-family conflict, in turn, is associated with employees’ negative health and well-being outcomes [14,15,16,17,18,19].

Based on the work-family interface model, previous studies have paid much attention to the negative work-to-family spillover effects of employees’ emotional labor, abusive supervision, and social ostracism at workplaces [10,11,12,13,20,21,22,23]. However, there has been lack of research examining the negative work-to-family spillover effects originate from workplace bullying. To address this gap in occupational literature, this study examines the potential mediating role of work-to-family conflict in the link between workplace bullying and employee well-being outcomes assessed by quality of life and occupational health. Most of existing studies on workplace bullying have been based on Western samples, lacking in consideration of different cultural values on interpersonal relationships or organizational hierarchies and cultures in non-Western countries [6,23,24]. Findings from the Korean employee sample may enrich our understanding of the mechanism in which workplace bullying impairs employee well-being in a cultural context where employees are particularly vulnerable to experiencing workplace bullying and work-to-family conflict.

### 1.1. Theoretical and Empirical Background Linking Workplace Bullying to Employee Well-Being

Workplace bullying is generally defined as situations where an employee is exposed to negative actions on the part of co-workers, supervisors or subordinates repeatedly and over a period of time [25]. It is different from workplace violence [26] or occupational stalking [27] in its nature of repetition, persistency, hostile intentionality of negative acts, and power imbalance. Some forms of workplace bullying behaviors include wrong or unjust judgement about a bullied employee’s work performance, criticizing one’s personal life, restricting expression of personal opinion, assigning meaningless tasks, and backbiting. Such negative actions are unwanted and resented by the victim employees and may cause humiliation and distress in victims and also potentially in observers [28].

Previous research has observed the negative consequences of workplace bullying on employees’ health and well-being, including deterioration of psychological well-being, complaints about physical and somatic symptoms, and poor quality of life [29,30,31,32]. Both the victims of bullying and the observers report more general stress and mental stress than those without bullying experiences [33]. There may also be a long-term health consequences of workplace bullying. A 3-wave follow-up study from Danish employees in a period of four years has shown that negative health problems caused by workplace bullying (e.g., poor self-rated health, sick-leave, depressive disorders, and sleep problems) last over several years even after bullying was discontinued [34].

### 1.2. Work-to-Family Conflict as a Mediating Mechanism

Work-family conflict refers to “*a form of interrole conflict in which the role pressures from work and family domains are mutually incompatible in some respect*” ([7], p. 77), which includes time-based, strain-based, and behavior-based conflict. The work-family interface model [7,8,9] suggests that negative experiences from work often spill over into employees’ non-work domains and interfere with family and personal activities (i.e., work-to-family conflict) that are critical for employee well-being. The emotional and strain-based work demands can threaten employees’ psychological resources including needs for autonomy, competence, and relatedness and hamper their involvement to meet role requirements in family and personal domains [35,36,37,38,39]. Through this work-to-family conflict mechanism, employees may transmit their negative emotions toward and come into conflict with family members, thereby their family roles, relationships, and family time may be negatively influenced [23,40]. Previous studies found the effects of work-to-family conflict on employee’s psychological distress [17,19], somatic symptoms and health complaints [14,15,16], and occupational well-being [18].

Many studies have examined work-to-family conflict consequences associated with employees’ emotional labor, non-supportive or abusive supervision, psychopathic leadership, and ostracism in workplaces [10,11,12,13,20,21,22,23]. Scant empirical research has been done on the work-to-family conflict effect on the link between workplace bullying and employee well-being outcomes such as quality of life and occupational health. Employees who are frequently exposed to workplace bullying may experience considerable strain at work in trying to defend and protect themselves. This consumption of victims’ physical and psychological resources might negatively spill over into their family and personal domains, which could impair well-being. One of the rare studies of this kind was recently performed by Sanz-Vergel and Rodríguez-Muñoz [41], who examined the mediating effect of work-to-family conflict on the relationship between workplace bullying and employees’ health problems in the telecommunications sector in Spain. They found that work-to-family conflict partially mediated the positive association between employee’s workplace bullying experiences and health problems including somatic symptoms, anxiety, and insomnia. Thus, based on the work-family interface model [7,8,9], we could propose that more exposure to workplace bullying is associated with lower well-being outcomes, mediated by higher work-to-family conflict.

### 1.3. Extent of Workplace Bullying in Korean Workplaces

Contextual characteristics in a certain culture and nation may influence on the people’s work and family life [42]. According to the well-known Hofstede’s cultural dimensions, Korea is considered to be a society with high levels of power distance, uncertainty avoidance, collectivism, Confucianism, and restraint [43,44]. In this culture, Korean workplaces have tended to have strong hierarchy of top-down organizational culture with the hard work ethic for long hours and let the group interests take precedence over the individual rights of employees [42,45,46], which is more likely to be a breeding ground for workplace bullying acts and behaviors [47]. For example, abusive supervisors or colleagues might exploit the victim’s work-oriented attitude by top-down leadership or collectivistic peer pressure.

According to Seo’s survey in 2010, 4% of Korean employees working in healthcare, manufacturing, service, and financial industries were the victims of workplace bullying and only 13.4% reported that they had never experienced any forms of workplace bullying during the past six months [4]. Among a number of Korean industries, employees working in education, banking, and healthcare industries seem more vulnerable; about 25% of education industry workers were the victims of workplace bullying and banking industry workers reported average 34 exposure to workplace bullying per month [5]. The most frequent negative acts experienced by the respondents were ‘being urged to resign’, ‘ideas or opinions being ignored’, and ‘being humiliated’. Especially, employees in education, banking, and healthcare sectors came under pressure to resign once a week. Employees who worked long hours or non-regular workers reported more exposure to workplace bullying [5].

Although workplace bullying is one of the major social problems in Korea and the media is paying attention to the recent suicide cases of employees due to severe stress from workplace bullying [48], this topic has received little scholarly attention. There has been lack of knowledge about the prevalence, antecedents, consequences, and mechanisms of bullying in Korean workplaces. To examine the associations between workplace bullying, work-to-family conflict, and employee well-being, the current study used data collected from employees in education, banking, and healthcare industries in Korea, where workplace bullying is a particular concern.

### 1.4. Present Study

**Figure 1:**
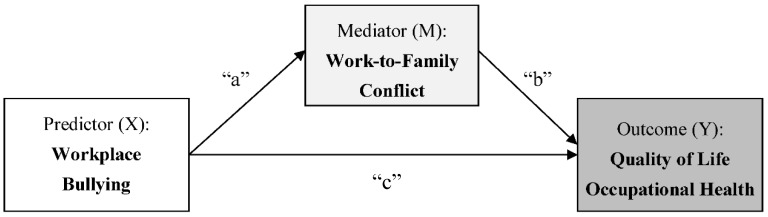
Conceptual model examining the effect of workplace bullying on employees’ well-being outcomes mediated by work-to-family conflict. **Note**: “a × b” indicates the indirect effect of X on Y through M. “c” indicates the total effect of X on Y.

Building on the work-family interface model [7,8,9], we examined the cross-sectional associations between workplace bullying, work-to-family conflict, and employees’ well-being outcomes. Using data collected from three service industries (i.e., healthcare, education, and banking) in Korea, we tested the mediating role of work-to-family conflict in the associations of workplace bullying with quality of life and occupational health, two outcomes reflecting employees’ overall well-being. Our hypotheses are as follows, with specific paths are illustrated in Figure 1.

**Hypotheses** **1.** *More exposure to workplace bullying will be associated with higher work-to-family conflict (“a” path)*.

**Hypotheses** **2.** *Higher work-to-family conflict will be associated with lower well-being, assessed by quality of life and occupational health (“b” path)*.

**Hypotheses** **3.** *More exposure to workplace bullying will be indirectly associated with lower well-being, mediated by higher work-to-family conflict (“a × b”)*.

## 2. Materials and Methods

### 2.1. Participants and Procedure

Employees working in healthcare, education, and banking industries in South Korea participated in this study. Participants were recruited across multiple worksites within each industry from July to September 2014. Those worksites included 4 clinics and hospitals (“healthcare” industry), 6 elementary, middle and high schools (“education” industry), and 12 banks, insurance companies, and other financial institutions (“banking” industry). All worksites were located in Seoul and Gyeonggi-do, the capital city and the province area surrounding the capital city, respectively. Only regular employees (full-time, permanent employees, not temporary) and middle managers and below level (not high-level and executives) were invited to participate in the study.

A paper-pencil questionnaire measuring respondent’s exposure to workplace bullying, work-to-family conflict, quality of life, occupational health, and demographic variables was administrated for about twenty minutes in the employee lounges, cafeterias, and lobbies at each workplace. Participants were briefed about the research purpose and requirements of this study, and then informed that their participation would be voluntary and anonymous, guaranteeing confidentiality. After they agreed to participate and provided consent, 444 questionnaires were distributed and 410 employees completed the survey, resulting in a high response rate of 92.3%. One of our main variables asked about the extent to which work experiences interfere with family and personal life (i.e., work-to-family conflict). Thus, we restricted our sample to those who were in heterosexual married/partnered status, because homosexual relationship is socially unacceptable and against the law in South Korea. Out of 410 employees who completed the questionnaire, 307 employees were heterosexual married/partnered, regular employees, and middle managers and below level at the time of survey, thus the final analytic sample of the current study. Their demographic information is provided in Table 1.

### 2.2. Measures

#### 2.2.1. Workplace Bullying

Exposure to workplace bullying was measured by twenty-two items of the Negative Acts Questionnaire (NAQ-Revised) [49]. Employees were asked to report the extent to which they had been exposed to specific negative behaviors at their workplace within the previous six months. Sample items include “Someone withholding information which affects your performance”, “Being ordered to do work below your level of competence”, and “Having your opinions and views ignored”. Each item was rated on a 5-point scale such as 0 = *never*, 1 = *now and then*, 2 = *monthly*, 3 = *every week*, and 4 = *daily*. Some previous studies considered a frequency of roughly weekly exposure over about 6 months as severe cases of workplace bullying [1]. To capture the effect of any exposure to workplace bullying in this study, we considered responses 1 or higher as *having exposure to workplace bullying* (=1 vs. 0 = *no exposure to workplace bullying*). Then we summed the binary indicators across 22 items to create total workplace bullying exposure variable; higher scores representing more exposure to workplace bullying. The Cronbach’s alpha for the 22 items was 0.92.

#### 2.2.2. Work-to-Family Conflict

Work-to-family conflict was measured with four items of the Work to Family Conflict Scale [50], in which employees were asked to report the extent to which they had experienced work conflicts with family in the past year. Each item was rated on a 5-point scale from 1 = *never* to 5 = *all of the time*. Sample items include “Your job reduces the effort you can give to activities at home”, “Stress at work makes you irritable at home”, and “Your job makes you feel tired to do the things that need attention at home.”. The mean of the 4 items was calculated, with higher scores representing greater work-to-family conflict. The Cronbach’s alpha for the 4 items was 0.82.

#### 2.2.3. Quality of Life

Employees’ perceptions of their quality of life were assessed via six items excerpted from the Quality of Life Scale-Parent Form [51]. Respondents rated their satisfaction in family life, time for work, family and leisure, and financial well-being on a 5-point Likert scale from 1 = *very dissatisfied* to 5 = *very satisfied*. Example items read, “How satisfied are you with your family life?”, “How satisfied are you with your time?”, and “How satisfied are you with your financial well-being?” The mean of the 6 items was calculated, with higher scores representing higher quality of life. The Cronbach’s alpha for the 6 items was 0.81.

#### 2.2.4. Occupational Health

To assess employees’ overall perceived health affected by their occupation, we used two items adapted from Zoller’s [52] interview question in terms of physical and psychological aspects. The items read, “How does your job affect your physical health?” and “How does your job affect your mental health?” Responses were coded as 1 = *very negatively*, 2 = *negatively*, 3 = *neither negatively nor positively*, 4 = *positively*, 5 = *very positively.* The mean of the two items was calculated, such that higher scores reflected greater occupational health.

#### 2.2.5. Covariates

We controlled for employees’ sociodemographic and work characteristics as covariates, including age, gender, education level, and work hours. Age and work hours as continuous variables were self-reported in years and hours, respectively. Gender (0 = *male*, 1 = *female*) and education level (0 = *under college graduate*, 1 = *college graduate or higher*) were dummy coded. In addition, we considered potential differences by industry. In our sample, the banking industry had the largest number of employees (see Table 1) and thus served as the reference group (1 = *healthcare*, 2 = *education*, 3 = *banking*; reference group).

### 2.3. Analytic Strategy

We used multiple mediation analyses with bootstrapping method using the SAS PROCESS macro [53]. This method allows for the estimation of the indirect effect, based on the product (×) of the effect of a predictor on a mediator and the effect of the mediator on an outcome. The indirect effect reflects “a × b” in Figure 1. The bootstrapping method also produces a bias-corrected confidence interval for the indirect effect [53]. In all models, we set the number of bootstrap samples to 10,000.

## 3. Results

Table 1 shows descriptive results of our variables and comparisons by industry. Beginning with sociodemographic characteristics, the average age of our sample was 42.85 years (*SD* = 8.01) and banking industry employees were older than healthcare industry employees (no difference with education industry employees). Sixty-one percent were women, with a higher proportion of women in the education industry (78%). The majority of the employees (70%) were college graduates or had higher education; this trend was more apparent in the education industry (91%) than in the banking industry (54%). The mean work hours was 43.83 h per week (*SD* = 6.22) and banking industry employees worked significantly longer hours than those in the other two industries.

In terms of our main variables, the mean exposure to workplace bullying for an average employee was not so high (*M* = 5.30 on a 0–22 range scale); yet, there was a great variability between employees (*SD* = 5.33). More than half of employees (54%) endorsed one particular item, “Someone withholding information which affects your performance.” Employees in the healthcare and banking industries reported significantly more exposure to workplace bullying than those in the education industry (with no difference between healthcare and banking). Our sample of employees reported a moderate level of work-to-family conflict (*M* = 2.97 on a 5 point scale) and a high level of quality of life (*M* = 3.62 on a 5 point scale), on average, with no differences by industry. The mean level of occupational health was moderate (*M* = 3.19 on a 5 point scale), and it was higher for education industry employees than for banking industry employees.

Table 2 shows results of the mediation model examining the effect of workplace bullying on quality of life through work-to-family conflict. The first column presents the results of “a” path, the association of workplace bullying with work-to-family conflict adjusting for covariates. Employees in the healthcare and education industries reported lower work-to-family conflict than those in the banking industry. Women (vs. men), employees with college or higher education (vs. not), and those with longer work hours reported higher work-to-family conflict. After controlling for these effects, there was a significant association of workplace bullying with work-to-family conflict, such that more exposure to workplace bullying was associated with higher work-to-family conflict. Moreover, higher work-to-family conflict was associated with lower quality of life (“b” path, second column). Before including work-to-family conflict, there was a significant negative association of workplace bullying with quality of life (“c” path; *B* = −0.034, *SE* = 0.007, *p* < 0.001); this association was slightly reduced after including work-to-family conflict (“c’” path; *B* = −0.027, *SE* = 0.007, *p* < 0.001). The association was found after adjusting for industry, sociodemographic characteristics, and work hours (none of them were significant). On the whole, then, the model revealed a significant indirect effect of workplace bullying on quality of life mediated by work-to-family conflict. Twenty percent of the total effect of workplace bullying on quality of life was explained by the indirect effect through work-to-family conflict.

Table 3 shows results of the mediation model examining the effect of workplace bullying on occupational health through work-to-family conflict. Consistent with the previous model (Table 2), more exposure to workplace bullying was associated with higher work-to-family conflict (“a” path). Further, higher work-to-family conflict was associated with lower occupational health (“b” path). This link was independent of the significant associations of education industry (vs. banking) and older age with higher occupational health. The total effect of workplace bullying on occupational health was also significant (“c” path; *B* = −0.031, *SE* = 0.009, *p* < .01). However, after including work-to-family conflict, the direct association of workplace bullying with occupational health was reduced (“c’” path; *B* = −0.018, *SE* = 0.009, *p* < 0.05). Overall, the model revealed a significant indirect effect of workplace bullying on occupational health mediated by work-to-family conflict. Forty-one percent of the total effect of workplace bullying on occupational health was due to the indirect effect through work-to-family conflict.

**Figure 2:**
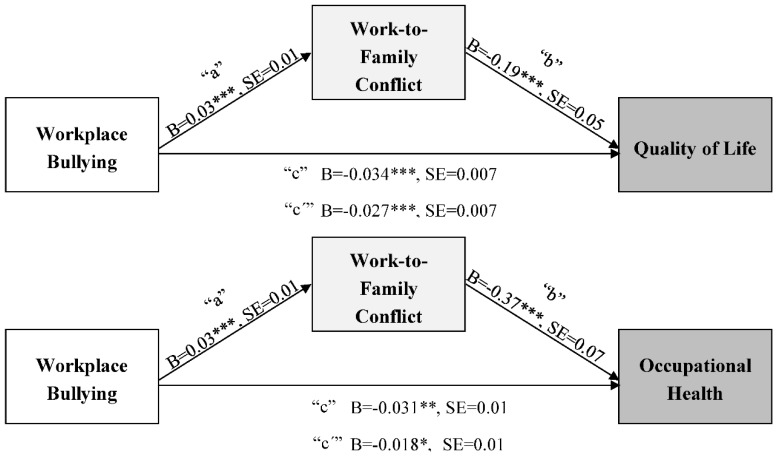
The mediating effects of work-to-family conflict on the links between workplace bullying on well-being outcomes. **Note**: Analyses adjusted for industry, age, gender, education, and work hours. “a × b” indicates the indirect effect of X on Y through M. “c” indicates the total effect of X on Y. “c’” indicates the direct effect of X on Y after controlling for the effect of M on Y.

Figure 2 summarizes our results showing the mediating effects of work-to-family conflict on the links between workplace bullying and two well-being outcomes. More exposure to workplace bullying was associated with higher work-to-family conflict (i.e., H1 supported), which was, in turn, associated with lower levels of quality of life and occupational health (i.e., H2 supported). Work-to-family conflict was a significant mediator in the association between workplace bullying and well-being (i.e., H3 supported).

## 4. Discussion

Guided by the work-family interface model [7,8,9], we examined the mediating role of work-to-family conflict in the associations between workplace bullying and well-being outcomes among Korean employees. Consistent with our hypotheses, results revealed that more exposure to workplace bullying was associated with greater work-to-family conflict, and greater work-to-family conflict was further associated with lower quality of life and occupational health. We have found no other studies that report the consequences and mechanisms of workplace bullying in Korean employees. Given that workplace bullying is a serious issue in many countries [3], our findings may add regional empirical evidence to the literature on workplace bullying.

We found that Korean employees who had more exposure to workplace bullying reported experiencing greater work-to-family conflict. This finding supports the work-family interface model [7,8,9] which suggests that stressful work experiences such as workplace bullying may spill over into employees’ non-work domains and interfere with family and personal activities. Specifically, stress from workplace bullying experiences might have threatened employees’ psychological resources and thus reduce their ability to be involved in family and personal roles and responsibilities [35,36,37,38,39]. Note that the mean levels of workplace bullying exposure and work-to-family conflict experiences were not high in our sample, but the two variables were positively covaried. It may also be important to mention differences in the levels of workplace bullying and work-to-family conflict by industry. We observed that Korean employees in the healthcare and banking industries reported significantly more exposure to workplace bullying than those in the education industry (see Table 1). Moreover, Korean employees in the healthcare and education industries reported higher work-to-family conflict than those in the banking industry after adjusting for sociodemographic characteristics and work hours (see Table 2). However, the positive association between workplace bullying and work-to-family conflict was found across the three industries, which may suggest the strong link between them.

Our results also revealed that greater work-to-family conflict was associated with lower levels of quality of life and occupational health. This is in line with previous studies that report the negative consequences of work-to-family conflict on employee health and well-being [14,15,16,17,18,19]. Korean employees work long hours and work in hierarchical culture [42,45,46], all of which may be risk factors for work-to-family conflict and degraded well-being. Given that happier employees are more productive at work [54], Korean employers should make more efforts to reduce work-to-family conflict and thereby improve their employees’ well-being. For example, a workplace intervention designed to increase supervisor support may reduce work-to-family conflict [55], and by doing so, improve employee health and well-being [14,56].

Combining these results, this study observed that workplace bullying was associated with employee well-being (i.e., quality of life, occupational health), and this association was partially mediated by work-to-family conflict. Before adding work-to-family conflict in our analytic models, workplace bullying was significantly associated with quality of life and occupational health. However, these associations became weaker after including work-to-family conflict. Although not fully mediated, considerable proportions in the total effects of workplace bullying on quality of life (24%) and occupational health (41%) were explained by work-to-family conflict. This study contributes to understanding the mechanisms in which workplace bullying is linked to Korean employees’ well-being. Future research may need to consider other potential mechanisms linking workplace bullying and employee well-being, as we found that work-to-family conflict did not fully mediate the association.

### 4.1. Practical Implications

Korea currently has no legal definition and laws on workplace bullying. This study urges that it’s about time to develop rules to reduce workplace bullying incidences in Korea as well as to protect Korean workers from its negative consequences. Most of European countries and parts of Canada and Australia have established laws and regulations against workplace bullying [57,58]. Their practices and success stories may guide Korean government’s legislation. In addition, work and life balance rather than achieving goals and career success is a continuously important topic among Korean employees because many Korean workplaces are highly competitive and demand individual sacrifice for the larger organization. The mediating effects of work-to-family conflict on the negative associations between workplace bullying and employee well-being found in this study suggest that each workplace needs to implement work-life balance policies and establish ethical standards and infrastructure [59] for the prevention and handling of workplace bullying. Workplace bullying may also involve substantial costs for the community due to degraded health as well as for the employers in terms of lost productivity. In order to legislate against workplace bullying in Korea, a business case needs to be made. Findings from this study may also provide broader implications for other countries who have similar issues of work and family life with Korea and want to improve their own workplace practices.

### 4.2. Limitations and Future Directions

Several of this study’s limitations provide useful directions for future research. First, we used self-reports of workplace bullying, work-to-family conflict, quality of life, and occupational health that may pose a risk for common-method bias [60]. For example, an employee who experienced more workplace bullying might have responded negatively to the items of quality of life and occupational health. Future research may benefit from incorporating objective measures of well-being, such as clinical health measures or biomarkers of stress. Second, our sample was purposely selected from multiple worksites in three industries (healthcare, education, and banking) in South Korea, and thus it is not representative of Korean employees. In the future, it is necessary to include workplace bullying items in a national survey so that we can draw national-level inference about the negative influence of workplace bullying. It may also be that our measure of workplace bullying may not fully capture the real phenomenon of workplace bullying. According to Seo [5], only about 38% of victim employees in Korea report the incidents of bullying, because of their perception that some extent of bullying is unavoidable in Korean workplace culture. As such, we may underestimate the extent of workplace bullying. Future research may need to improve the validity of workplace bullying measure by cultural and occupational contexts. More specific measurements about workplace bullying are also needed. For example, there may be differences between men and women in the experience of workplace bullying consequences of it [61]. Moreover, more regional analyses are needed to see whether findings from our study are replicated in other settings. Finally, our cross-sectional analyses cannot determine the direction of effect. Although our analytic models imply that workplace bullying is a predictor, work-to-family conflict is a mediator, and quality of life and occupational health are outcomes, there is no temporal order between the variables and causality can operate in other directions. Future research should include multiple time points to identify the direction of effect.

## 5. Conclusions

Findings from this study highlight that workplace bullying is an important work-derived stressor associated with Korean employees’ work-to-family conflict and well-being outcomes. All of our research hypotheses were supported: More exposure to workplace bullying was associated with lower levels of quality of life and occupational health among Korean employees; specifically, the negative associations were mediated by greater work-to-family conflict. At the most basic level, both workplace bullying and work-to-family conflict are societal concerns, and thus future research should continue to focus on this topic by examining multiple pathways linking workplace bullying to well-being outcomes in diverse employee samples across countries. A more harmonious workplace may improve the employees’ well-being, which may ultimately enhance productivity and health at the larger society.

## Figures and Tables

**Table 1:** Descriptive statistics of variables by sub-industry.

	Total (*N* = 307)		Healthcare (*n* = 105)		Education (*n* = 88)		Banking (*n* = 114)		Difference Test
	*M* or %	(*SD*)	*M* or %	(*SD*)	*M* or %	(*SD*)	*M* or %	(*SD*)	*χ*^2^ or *F-test*
*Sociodemographic characteristics*									
Age	42.85	(8.01)	41.28_b_	(7.99)	43.07_ab_	(8.52)	44.14_a_	(7.41)	3.60 *
Gender (%)									15.41 ***
Male	38.76		46.67		21.59				
Female	61.24		53.33		78.41		44.74		
Education (%)							55.26		31.59 ***
College graduate or higher	70.03		69.52		90.91		54.39		
Under college graduate	29.97		30.48		9.09		45.61		
Work hours (per week)	43.83	(6.22)	43.34_b_	(4.44)	41.89_b_	(4.00)	45.78_a_	(8.20)	10.89 ***
*Main variables*									
Workplace bullying	5.30	(5.33)	6.00_a_	(5.56)	3.64_b_	(3.95)	5.95_a_	(5.77)	6.25 **
Work-to-family conflict	2.97	(0.79)	2.88	(0.86)	2.94	(0.76)	3.07	(0.73)	1.58
Quality of life	3.62	(0.61)	3.58	(0.60)	3.74	(0.61)	3.55	(0.61)	2.70
Occupational health	3.19	(0.88)	3.16_ab_	(0.94)	3.44_a_	(0.79)	3.03_b_	(0.84)	5.89 **

Note: *N* = 307 Korean employees. Differing subscripts of a, b, and c indicate the results of post hoc analyses where a is higher than b (ab is not significantly different from a or b). Means and percentages with no subscripts do not significantly differ. * *p* < 0.05, ** *p* < 0.01, *** *p* < 0.001.

**Table 2:** The effect of workplace bullying on quality of life, mediated by work-to-family conflict.

	**M: Work-to-Family Conflict**			**Y: Quality of Life**		
	***B***		**(*SE*)**	***B***		**(*SE*)**
Intercept	−0.45	***	(0.10)	3.55	***	(0.08)
**X**: Workplace bullying	0.03	***	(0.01)	−0.03	***	(0.01)
**M**: Work-to-family conflict	--		--	−0.19	***	(0.05)
Industry, Healthcare (vs. Banking)	−0.22	*	(0.10)	−0.02		(0.08)
Industry, Education (vs. Banking)	−0.26	*	(0.11)	0.08		(0.09)
Age	−0.01		(0.01)	0.00		(0.00)
Women (vs. Men)	0.54	***	(0.09)	0.01		(0.07)
College graduates or higher (vs. Not)	0.38	***	(0.10)	0.07		(0.08)
Work hours (per week)	0.01	*	(0.01)	0.00		(0.01)
	*R*^2^ = 0.2285			*R*^2^ = 0.1553		
	*F*(7299) = 12.65 ***			*F*(8298) = 6.85 ***		
	Indirect Effect of X on Y: B = −0.01 **, *SE* = 0.002 95% CI = −0.0121 to −0.0129					

Note: *N* = 307 Korean employees. X refers to predictor; M refers to mediator; Y refers to outcome. * *p* < 0.05, *** *p* < 0.001.

**Table 3:** The effect of workplace bullying on occupational health, mediated by work-to-family.

	**M: Work-to-Family Conflict**			**Y: Occupational Health**		
	***B***		**(*SE*)**	***B***		**(*SE*)**
Intercept	−0.45	***	(0.10)	3.04	***	(0.12)
**X:** Workplace bullying	**0.03**	***	**(0.01)**	**−0.02**	*	**(0.01)**
**M:** Work-to-family conflict	--		--	**−0.37**	***	**(0.07)**
Industry, Healthcare (vs. Banking)	−0.22	*	(0.10)	0.13		(0.11)
Industry, Education (vs. Banking)	−0.26	*	(0.11)	0.36	**	(0.13)
Age	−0.01		(0.01)	0.02	**	(0.01)
Women (vs. Men)	0.54	***	(0.09)	0.03		(0.10)
College graduates or higher (vs. Not)	0.38	***	(0.10)	−0.03		(0.11)
Work hours (per week)	0.01	*	(0.01)	0.00		(0.01)
	*R*^2^ = 0.2285			*R*^2^ = 0.2035		
	*F*(7299) = 12.65 ***			*F*(8298) = 9.52 ***		
	Indirect Effect of X on Y: *B* = −0.01***, *SE* = 0.004 95% CI = −0.0214 to −0.0064					

Note: *N* = 307 Korean employees. * *p* < 0.05, ** *p* < 0.01, *** *p* < 0.001.
